# ﻿Catalogue of primary types of Neotropical *Myotis* (Chiroptera, Vespertilionidae)

**DOI:** 10.3897/zookeys.1105.85055

**Published:** 2022-06-15

**Authors:** Roberto Leonan M. Novaes, Don E. Wilson, Ricardo Moratelli

**Affiliations:** 1 Fundação Oswaldo Cruz, Fiocruz Mata Atlântica, R. Sampaio Correa s/n, Taquara, 22713-560, Rio de Janeiro, Brazil Fundação Oswaldo Cruz, Fiocruz Mata Atlântica Rio de Janeiro Brazil; 2 Smithsonian Institution, National Museum of Natural History, Division of Mammals. 10th St. & Constitution Ave. NW, 20013-7012, Washington, DC, USA National Museum of Natural History Washington, DC United States of America

**Keywords:** Myotinae, taxonomy, type locality, type specimen, vespertilionid bats, zoological nomenclature, espécime-tipo, localidade-tipo, morcego vespertilionídeo, Myotinae, nomenclatura zoológica, taxonomia

## Abstract

*Myotis* comprises a diverse group of vespertilionid bats with worldwide distribution. Neotropical *Myotis* have an accentuated phenotypic conservatism, which makes species delimitation and identification difficult, hindering our understanding of the diversity, distribution, and phylogenetic relationships of taxa. To encourage new systematic reviews of the genus, a catalogue of the primary types and names is presented, current and in synonymy, for Neotropical *Myotis*. Currently 33 valid species (and three subspecies) are recognized, and their primary types are deposited in 12 scientific collections in the USA (30 types), Brazil (two types), England (two types), and France (one type). The names of 29 Neotropical *Myotis* species currently in synonymy were found. However, it is possible that some synonyms represent independent evolutionary lineages, considering recent results provided by taxonomic revisions.

## Abstract (pt)

*Myotis* compreende um grupo diverso de morcegos vespertilionídeos amplamente distribuídos ao redor do planeta. *Myotis* neotropicais têm um acentuado conservatismo fenotípico, o que dificulta a delimitação e identificação de espécies a partir de caracteres morfológicos, criando barreiras para a compreensão da diversidade, distribuição e relações filogenéticas entre os táxons. Visando encorajar novas revisões sistemáticas para o gênero, é apresentado este catálogo de nomes e tipos primários, válidos e sob sinonímia, para *Myotis* neotropicais. Atualmente, são reconhecidas 33 espécies válidas (e três subespécies) e seus tipos estão depositados em 12 coleções científicas nos EUA (30 tipos), Brasil (2 tipos), Inglaterra (2 tipos) e França (1 tipo). Compilamos 29 nomes de espécies de *Myotis* atualmente sob sinonímia. Entretanto, é possível que alguns desses sinônimos possam representar linhagens evolutivas independentes, considerando resultados recentes de revisões taxonômicas.

## ﻿Introduction

Taxonomy is the discipline of Biology responsible for describing, classifying, and naming organisms, as well as hypothesize about the evolutionary relationships between taxa (Tancoigne et al. 2011). Therefore, understanding and organizing biological diversity is the primary task of the taxonomist. Taxonomic studies have profound implications in virtually all areas of the biological sciences, such as ecology, evolution, genetics, and epidemiology, in addition to directly influencing public policies focused on health and environment (Cracraft 2002; Pearson et al. 2011; Cook et al. 2020). Furthermore, knowing the real diversity of organisms on our planet is critical for the sustainable use of natural resources and for the management and conservation of species (May 1988), especially in the current biodiversity crisis, where the rate of extinction indicates that we are witnessing a sixth mass extinction (Ceballos et al. 2015, 2017).

Species are the central unit of taxonomy and the association between an unambiguous scientific name and a species is of paramount importance for a reliable biological information system (Wheeler 2004). For that, the existence of primary types, which are those specimens designated as the name-bearing representative of a species is essential. In addition to serving as a reference point for the existence of any organism, type-specimens are a particularly important source of information for scientists to track and unravel the taxonomic history of biologically complex groups, such as bats of the genus *Myotis* Kaup, 1829.

*Myotis* is the most speciose genus of bats and the second largest genus of mammals, with more than 140 extant species (MDD 2021). It is also the genus with the greatest area of distribution among non-human mammals (Moratelli et al. 2019a). The greatest diversity and abundance of *Myotis* is reported in temperate and subtropical areas (Nowak 1994; Moratelli et al. 2019a). However, recent systematic reviews have indicated that there is a high diversity of *Myotis* in the Neotropics (e.g., Larsen et al. 2012; Moratelli et al. 2011a, 2013, 2016, 2017, 2019b; Carrión-Bonilla and Cook 2020; Novaes et al. 2021a, b, c). Nevertheless, our knowledge of species limits, name validity, and distributional boundaries for several Neotropical *Myotis* species remains incipient.

Part of the taxonomic hurdle is due to the accentuated morphological conservatism and lack of specimen series covering all geographic distributions (Menu 1987; Smith et al. 2012; Moratelli et al. 2019a). On the other hand, molecular studies have revealed the existence of more independent evolutionary lineages than species recognized from morphology-based taxonomy (Larsen et al. 2012; Novaes et al. 2021a, b). This indicates the existence of hidden diversity possibly composed of multiple cryptic species, which challenges the delimitation of species and raises the need for new systematic reviews, especially those based on multiple lines of evidence.

To contribute to the organization of systematic knowledge about this genus, and to support future studies of taxonomy, we present a catalogue of the primary types of Neotropical *Myotis*. Later, we briefly comment on the validity and distribution of some species.

## ﻿Materials and methods

The catalogue was mostly compiled by analysis of the specimens deposited in 12 biological collections: American Museum of Natural History (New York, USA), Field Museum of Natural History (Chicago, USA), Louisiana State University Museum of Natural Science (Baton Rouge, USA), Museum of Texas Tech University (Lubbock, USA), Museum of Vertebrate Zoology at University of California (Berkeley, USA), Kansas University Biodiversity Institute and Natural History Museum (Lawrence, USA), Natural History Museum, Los Angeles County (Los Angeles, USA), Smithsonian’s National Museum of Natural History (Washington D.C., USA), Natural History Museum, London (London, UK), Zoologisches Staats-Sammlung München (Munich, Germany), Muséum National D’Histoire Naturelle (Paris, France), Muséum d’Histoire Naturelle (Geneva, Switzerland), Museu de Zoologia da Universidade de São Paulo (São Paulo, Brazil), Universidade Federal Rural do Rio de Janeiro (Seropédica, Brazil). When it was not possible to visit the collection to examine the type specimen, the information was retrieved from the original species descriptions or other available bibliography (e.g., LaVal 1973; Carter and Dolan 1978; Carrión-Bonilla and Cook 2020) and by direct consultation with the curators of the collections. Abbreviations of biological collections cited in the text are available below.

**ALP**Universidade Federal Rural do Rio de Janeiro, Seropédica, Brazil;

**ANSP**Academy of Natural Sciences of Drexel University, Philadelphia, USA;

**AMNH**American Museum of Natural History, New York, USA;

**BMNH**Natural History Museum, London, UK;

**FMNH**Field Museum of Natural History, Chicago, USA;

**KU** Natural History Museum, Kansas University, Lawrence, USA;

**LACM**Natural History Museum, Los Angeles County, Los Angeles, USA;

**LSU**Louisiana State University Museum of Natural Sciences, Baton Rouge, USA;

**MHNG**Muséum d’Histoire Naturelle, Geneva, Switzerland;

**MNHN**Muséum National D’Histoire Naturelle, Paris, France;

**MSB**Museum of Southwestern Biology, University of New Mexico, Albuquerque, USA;

**MVZ**Museum of Vertebrate Zoology, University of California, Berkeley, USA;

**MZUSP**Museu de Zoologia da Universidade de São Paulo, São Paulo, Brazil;

**RNH** Rijksmuseum van Natuurlijke Historie, Leiden, Netherlands;

**TTU**Museum of Texas Tech University, Lubbock, USA;

**USNM**Smithsonian’s National Museum of Natural History, Washington D.C., USA;

**ZSM**Zoologisches Staats-Sammlung München, Munich, Germany.

The list of *Myotis* species adopted here is based on systematic reviews conducted for the genus *Myotis* in the Neotropical region (i.e., LaVal 1973; Bogan 1978; Moratelli et al. 2019a, b; Carrión-Bonilla and Cook 2020; Novaes et al. 2021a, b, c). Following LaVal (1973), we excluded species from the definition of Neotropical *Myotis* when their distributions extend from the Nearctic into the Neotropics. Geographical coordinates of type localities were retrieved, when available, directly from the original publications or by consulting the museum database and the gazetteer of Gardner (2008). In cases where they were not available, we used proximal coordinates of the locality from the search in the USA’s National Geospatial – Intelligence Agency (https://geonames.nga.mil/namesgaz/). We follow the International Code of Zoological Nomenclature (ICZN 1999) as a reference for the categories of type specimens.

The list of names was divided in two parts, the first with accounts of name-bearing type specimens of currently recognized species; and the second with accounts of name-bearing type specimens in synonymy. The accounts were arranged chronologically, following the date of taxa description. The format of accounts was inspired by Fisher and Ludwig (2015), but with modifications. Each account reads as follows: (i) Current name (for recognized species) or original published name (for names in synonymy) followed by the author’s or authors’ names; (ii) Original citation, including publication, volume, pages, and year of publication; (iii) Taxonomy, species original published name if different from the currently name, followed by information on subspecies, if any; (iv) Type designation as holotype, lectotype, paralectotype, neotype, or syntype, including collection number, age and sex, date collected and collector(s) name(s), and preparation of specimen; (v) Type locality: Verbatim locality as given in the original description or neotype designation, published restrictions, and supplementary data. Abbreviations are used for miles (mi), kilometers (km), feet (ft), and meters (m); (vi) Remarks, with additional information is provided as needed, but especially to explain types designated subsequent to description.

## ﻿Results

### ﻿Name-bearing type specimens of recognized species

**Figure 1.**
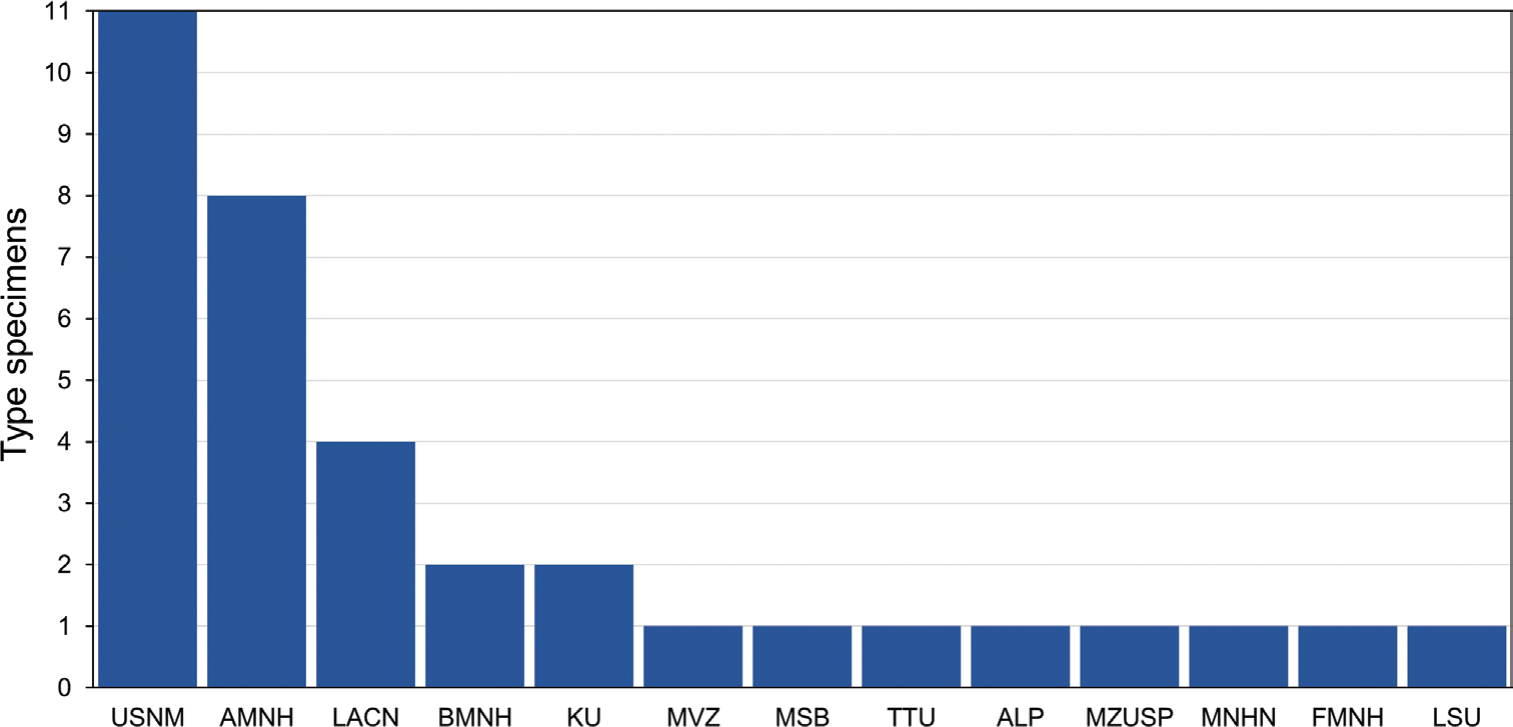
Number of primary type specimens of Neotropical *Myotis* deposited in zoological collections. The name of the institution for each acronym shown in the graph is described in the methods.

For the 33 species (and three subspecies) of Neotropical *Myotis* currently recognized (Table 1), primary types are deposited in 12 zoological collections in the USA (eight collections), Brazil (two collections), England (one collection) and France (one collection). The USA is home to 30 primary types of Neotropical *Myotis*, while Brazil and England are home to two primary types each and France to one type specimen. The collections with the largest number of primary types are the Smithsonian’s National Museum of Natural History (11 types), followed by the American Museum of Natural History (eight types), both in the USA. The other collections have 1–4 type specimens each (Fig. 1).

**Table 1.** Valid species and subspecies of Neotropical *Myotis* including information on their primary types.

Species	Type specimen	Proximal type locality
*M.albescens*	Neotype AMNH 205195	Paraguarí, Paraguay
*M.armiensis*	Holotype MSB 262089	Chiriquí, Panamá
*M.atacamensis*	Neotype USNM 391786	Tarapacá, Chile
*M.attenboroughi*	Holotype USNM 540693	St. John Parish, Tobago Island
*M.bakeri*	Holotype MVZ 136907	Lima, Peru
*M.carteri*	Holotype LACM 36876	Jalisco, Mexico
*M.caucensis*	Holotype AMNH 32787	Valle del Cauca, Colombia
*M.chiloensis*	Neotype FMNH 24029	Chiloé Island, Chile
*M.clydejonesi*	Holotype TTU 109227	Sipaliwini, Suriname
*M.cobanensis*	Holotype AMNH 145017	Alta Verapaz, Guatemala
*M.diminutus*	Holotype USNM528569	Los Ríos, Ecuador
*M.dinellii*	Holotype BMNH 0.7.9.4	Tucumán, Argentina
*M.dominicensis*	Holotype USNM 113564	Dominica
*M.elegans*	Holotype KU 88398	Veracruz, Mexico
*M.findleyi*	Holotype USNM 512417	Islas Tres Marías, Mexico
*M.handleyi*	Holotype USNM 370932	Distrito Federal, Venezuela
*M.izecksohni*	Holotype ALP 6675	Rio de Janeiro, Brazil
*M.keaysi*	Holotype AMNH 15814	Puno, Peru
*M.larensis*	Holotype AMNH 130709	Lara, Venezuela
*M.lavali*	Holotype MZUSP 18762	Pernambuco, Brazil
*M.levis*	Syntype MNHN 1997-1805	Southern Brazil
*M.martiniquensis*	Holotype AMNH 214062	Tartane, Martinique
*M.midastactus*	Holotype AMNH 211156	Beni, Bolívia
*M.moratellii*	Holotype USNM 513482	Los Ríos, Ecuador
*M.nesopolus*	Holotype USNM 101849	Curaçao, Netherlands Antilles
*M.n.nigricans*	Neotype LACN 36877	Rio de Janeiro, Brazil
*M.n.extremus*	Holotype USNM 77670	Chiapas, Mexico
*M.n.osculati*	Not located	Eastern Ecuador
*M.nyctor*	Holotype KU 109473	St. Thomas Parish, Barbados
*M.o.oxyotus*	Neotype LACN 36878	Carchi, Ecuador
*M.o.gardneri*	Holotype LSU 12924	San José, Costa Rica
*M.pampa*	Holotype AMNH 205471	Artigas, Uruguay
*M.pilosatibialis*	Holotype LACN 36879	Francisco Morazán, Honduras
*M.riparius*	Holotype USNM 310255	Darién, Panamá
*M.ruber*	Neotype USNM 115097	Paraguarí, Paraguay
*M.simus*	Holotype BMNH 8.5.12.2	Loreto, Peru

**Figure 2.**
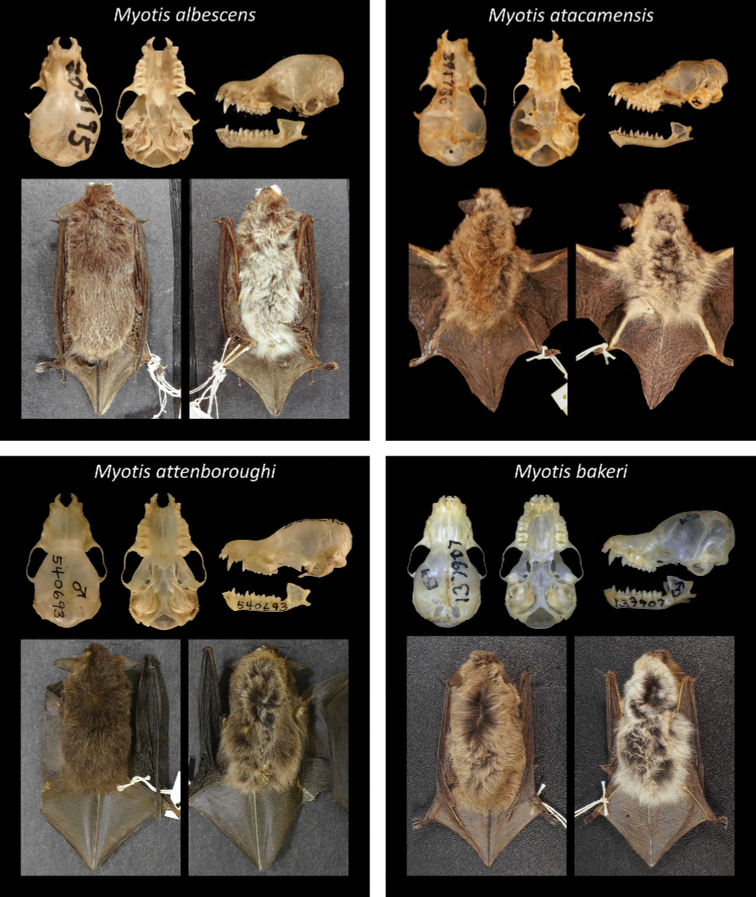
Some type specimens from valid species of Neotropical *Myotis*: AMNH 205195, neotype of *M.albescens*; USNM 391786, neotype of *M.atacamensis*; USNM 540693, holotype of *M.attenboroughi*; MVZ 136907, holotype of *M.bakeri*.

**Figure 3.**
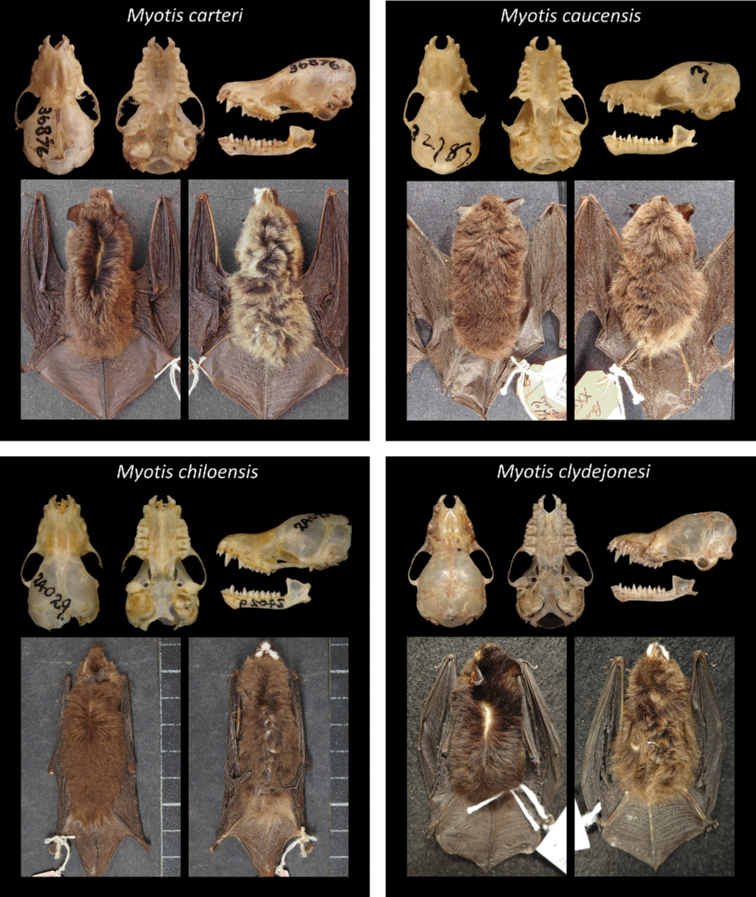
Some type specimens from valid species of Neotropical *Myotis*: LACM 36876, holotype of *M.carteri*; AMNH 32787, holotype of *M.caucensis*; FMNH 24029, neotype of *M.chiloensis*; TTU 109227, holotype of *M.clydejonesi*.

**Figure 4.**
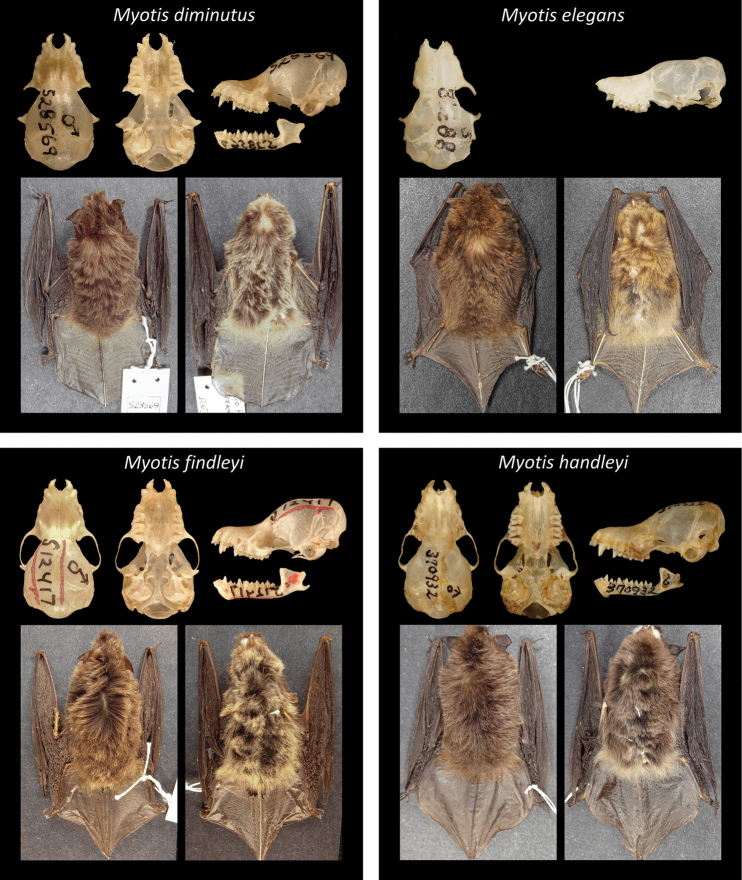
Some type specimens from valid species of Neotropical *Myotis*: USNM528569, holotype of *M.diminutus*; KU 88398, holotype of *M.elegans*; USNM 512417, holotype of *M.findleyi*; USNM 370932, holotype of *M.handleyi*.

**Figure 5.**
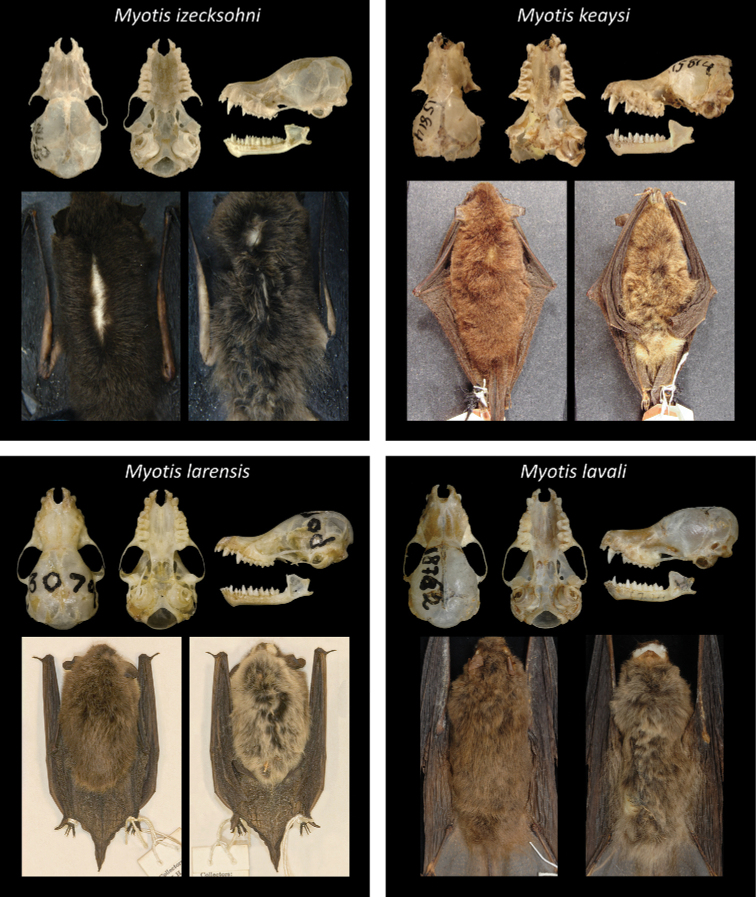
Some type specimens from valid species of Neotropical *Myotis*: ALP 6675, holotype of *M.izecksohni*; AMNH 15814, holotype of *M.keaysi*; AMNH 130709, holotype of *M.larensis*; MZUSP 18762, holotype of *M.lavali*.

**Figure 6.**
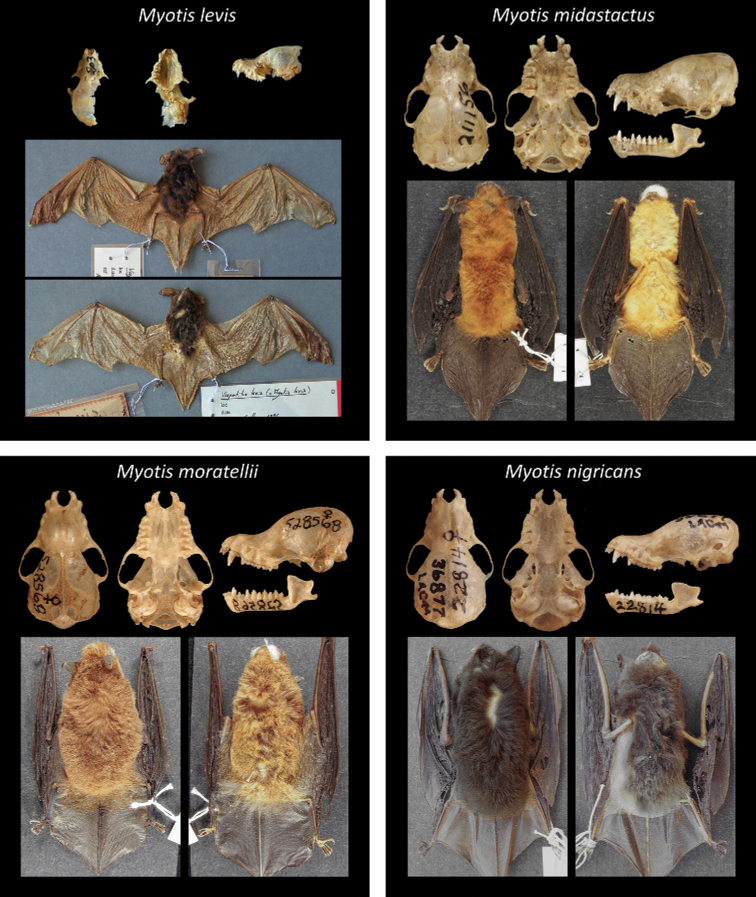
Some type specimens from valid species of Neotropical *Myotis*: MNHN 1997-1805, syntype of *M.levis*; AMNH 211156, holotype of *M.midastactus*; USNM 513482, holotype of *M.moratellii*; LACN 36877, neotype of *M.nigricans*.

**Figure 7.**
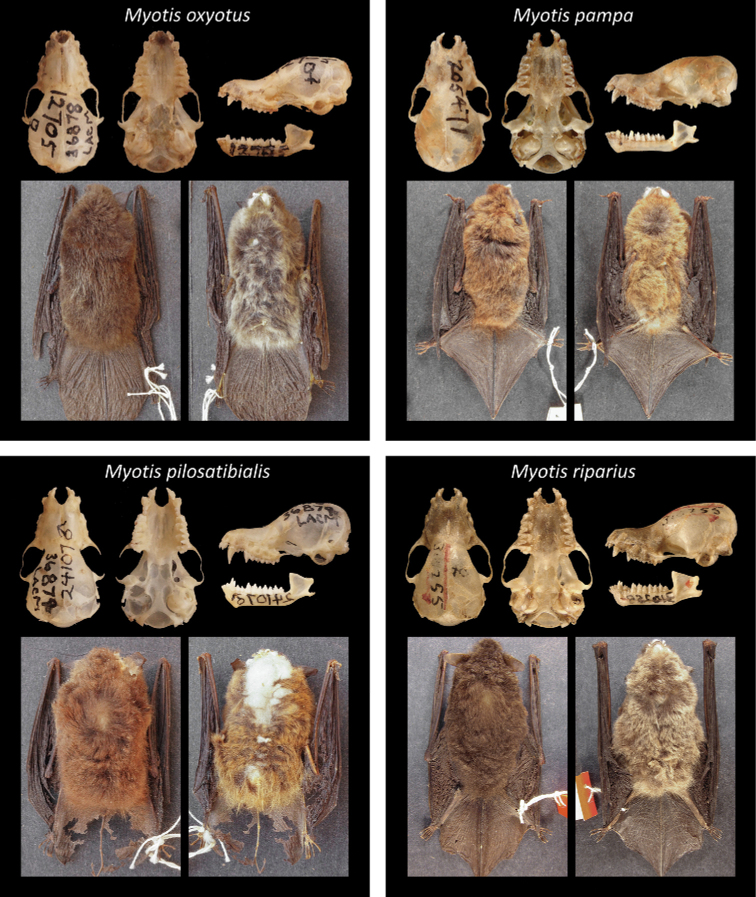
Some type specimens from valid species of Neotropical *Myotis*: LACN 36878, neotype of *M.oxyotus*; AMNH 205471, holotype of *M.pampa*; LACN 36879, holotype of *M.pilosatibialis*; USNM 310255, holotype of *M.riparius*.

**Figure 8.**
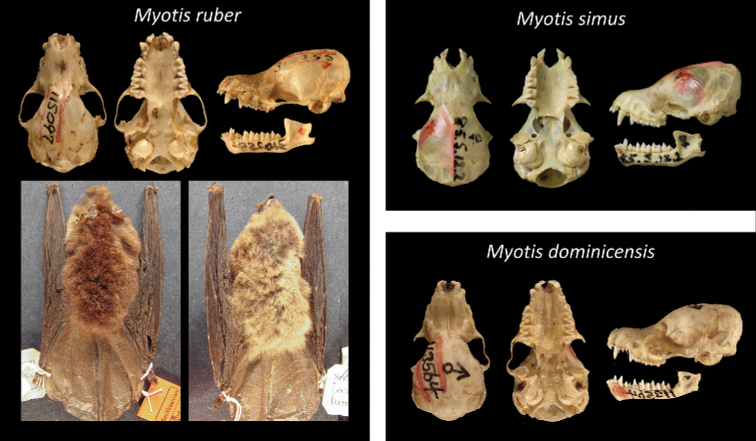
Some type specimens from valid species of Neotropical *Myotis*: USNM 115097, neotype of *M.ruber*; BMNH 8.5.12.2, holotype of *M.simus*; USNM 113564, holotype of *M.dominicensis*.

Of all recognized Neotropical *Myotis* types, 28 are holotypes, six are neotypes, and one is syntype. Only one taxon lacks a type specimen (*M.nigricansosculati*), which presumably was destroyed. About 95% of the type specimens are preserved as skin and skull, with mandible; while only 5% are preserved in fluid (usually alcohol 70°GL). Most types (80%) are in a good condition, with complete skulls and untorn skin. The other 20% are damaged, especially the oldest ones. Damages include broken skulls, loss of bone elements, or torn skins (Figs 2–8).

**Figure 9.**
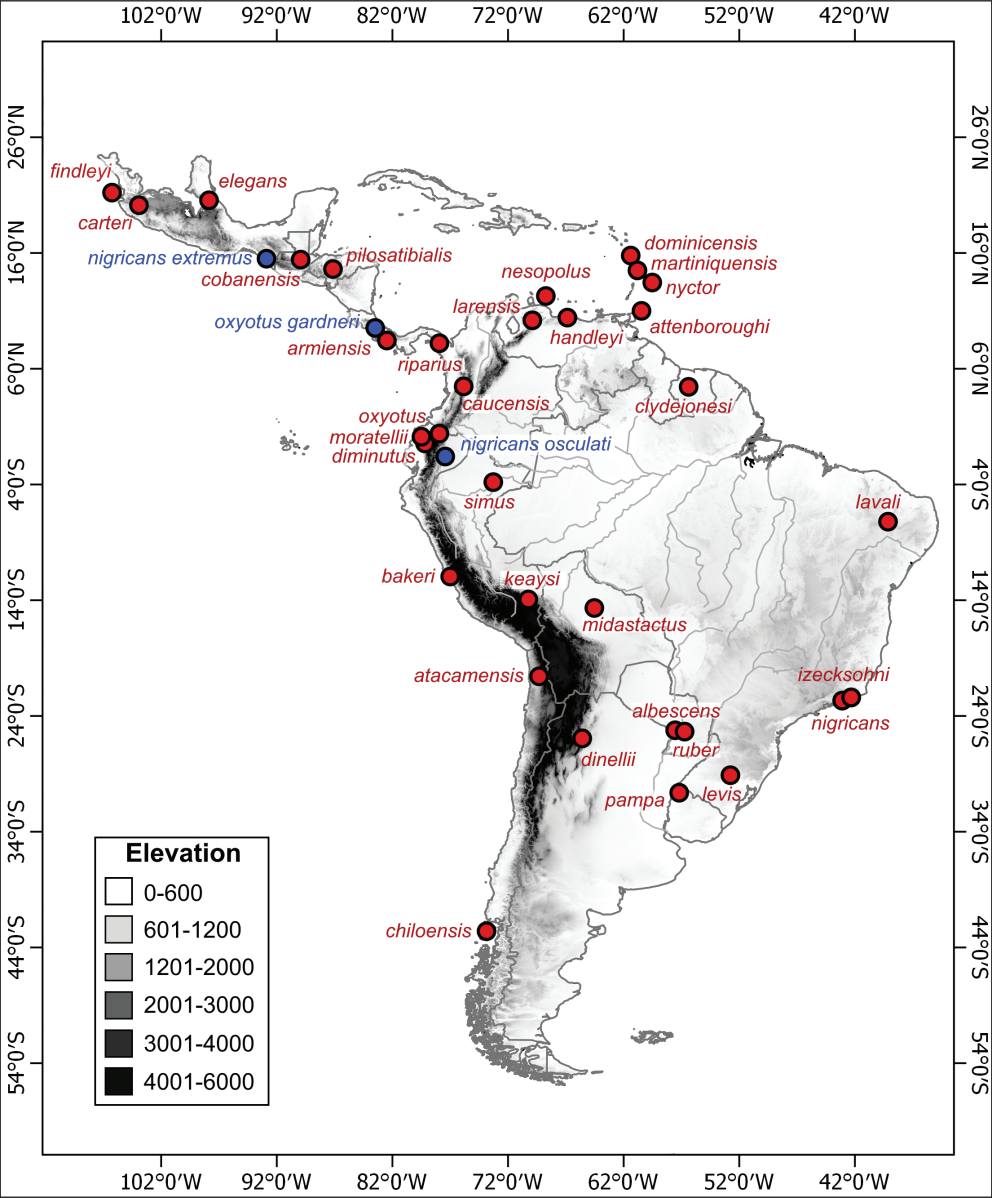
Type localities of the currently valid species (red) and subspecies (blue) of Neotropical *Myotis*.

Below is an annotated list of Neotropical *Myotis* species (organized in chronological order of description), with information about the primary specimen types and the type locality. We include a map containing the geographical point of all type localities (Fig. 9).

***Myotisruber* (É. Geoffroy, 1806)**

Annales du Muséum d’Histoire Naturelle 8: 187–205.

**Taxonomy**: Described as *Vespertilioruber* by Geoffroy Saint-Hilaire (1806: 204). Treated as monotypic (Wilson 2008; Moratelli et al. 2019a).

**Neotype**: USNM 115097, adult male collected on May 22, 1901 by W. Foster; skull, mandible, and skin.

**Type locality**: Sapucay (= Sapucai, Paraguarí), Paraguay (25°40'S, 56°57'W; ca. 200 m a.s.l.) by neotype designation (LaVal 1973: 45).

**Remarks**: The holotype was not specified by the author, who based his description on the Azara’s (1801) “chauve-sourris cannelle”. LaVal (1973) noted that D. C. Carter did not locate the specimen at the Muséum National d’Histoire Naturelle (Paris, France) or in any other European museum, concluding that it was lost or destroyed. The neotype was designated by LaVal (1973: 45), following the same reasoning presented for *M.albescens* (see below).

***Myotisalbescens* (É. Geoffroy, 1806)**

Annales du Muséum d’Histoire Naturelle 8: 187–205.

**Taxonomy**: Described as *Vespertilioalbescens* by Geoffroy Saint-Hilaire (1806: 204–205). Treated as monotypic (Moratelli and Oliveira 2011; Moratelli et al. 2019a).

**Neotype**: AMNH 205195, adult female collected on June 2, 1963 by M. D. Tuttle; skull, mandible, complete post-cranial skeleton and skin.

**Type locality**: Yaguarón, Paraguarí, Paraguay (25°33'S, 57°17'W; ca. 200 m a.s.l.) based on neotype designation (LaVal 1973: 26).

**Remarks**: The holotype was not specified by the author, who based his description on the Azara’s (1801) “chauve-souris donzième”. LaVal (1973) noted that D. C. Carter was unable to locate a specimen in European zoological collections from Azara’s expedition. According to Cabrera (1958), É. Geoffroy based his description of *Phyllostomalineatum* (currently *Platyrrhinuslineatus*) on specimens collected in Paraguay and listed by Azara (1801) in the same publication in which he listed the specimen that Geoffroy described as *Vespertilioalbescens*. Cabrera stated that the specimen of *P.lineatum* was destroyed. LaVal (1973: 26) considered that the type specimen of *V.albescens* met the same fate and designated a neotype.

***Myotisnigricans* (Schinz, 1821)**

**Taxonomy**: Originally *Vespertilionigricans* Schinz, 1821. We follow Bogan (1978) and Moratelli et al. (2019a) in recognizing three subspecies, and in treating *M.carteri* as a distinct species, instead of a subspecies of *M.nigricans*.

***Myotisnigricansnigricans* (Schinz, 1821)**

In “Das tierreich eingetheilt nach dem Bau der thiere als Grundlage ihrer Naturgeschichte und der vergleichenden Anatomie von dem Herrn Ritter von Cuvier, volume 1”. Saugethiere und Vögel, Stuttgart and Tübingen, 894 pp.

**Taxonomy**: Described as *Vespertilionigricans* by Schinz (1821: 179).

**Neotype**: LACM 36877, adult female collected on October 14, 1968 by A. L. Peracchi; skull, mandible, and skin.

**Type locality**: Seropédica, 42 km S Rio de Janeiro, Brazil (22°45'S, 43°41'W; 33 m a.s.l.).

**Remarks**: The species was described based on a specimen collected by Prinz Maxililian zu Wied-Neuwied at Fazenda do Agá, near the Rio Iritiba, Espírito Santo, Brazil. Miller and Allen (1928) were not able to confirm the existence of this specimen. From a personal communication of D. C. Carter, that he could not locate it in among the important collection of Wied’s Brazilian specimens at the American Museum of Natural History, LaVal (1973: 9) presumed it has been lost and designated a neotype.

***Myotisnigricansosculati* (Cornalia, 1849)**

In “Vertebratorum synopsis in Museo Mediolanense extantium que per novam orbem Cajetanus Osculati collegit annis 1846–47–1848 (…)”. Typographia Corbetta, Modoetiae, 16 pp.

**Taxonomy**: Described as *Vespertilioosculati* by Cornalia (1849: 11).

**Type specimen**: None. The specimen used in the original description was collected by G. Osculati between 1846 and 1848. Osculati’s collection deposited in the Museo di Storia Naturale di Milano, Italy, in 1848, was destroyed during World War II (Cagnolaro and Violani 1988; Moratelli et al. 2013).

**Type locality**: Eastern Ecuador.

***Myotisnigricansextremus* Miller & Allen, 1928**

Bulletin of the United States National Museum 144: 1–218.

**Taxonomy**: Described as *Myotisnigricansextremus* by Miller and Allen (1928: 181).

**Holotype**: USNM 77670, adult female collected by the E. W. Nelson and E. A. Goldman on March 1, 1896; skull, mandible, and skin.

**Type locality**: Huehuetán, Chiapas, Mexico (15°01'N, 92°22'W; 91 m a.s.l.).

***Myotislevis* (I. Geoffroy, 1824)**

Annales de Sciences Naturelles de Paris 3: 440–447.

**Taxonomy**: Described as *Vespertiliolevis* by Geoffroy Saint-Hilaire (1824: 444); currently monotypic (Moratelli et al. 2019a).

**Syntype**: MNHN type 203 (also referred to as MNHN 1997-1805), adult, sex unknown, collected by A. Geoffroy Saint-Hilaire (date not specified); mounted specimen, with skull removed and severely damaged.

**Type locality**: Southern Brazil.

***Myotischiloensis* (Waterhouse, 1840)**

In “The zoology of the voyage of the H.M.S. Beagle, under the command of Captain Fitzroy, R.N., during the years 1832 to 1836”. Smith, Elder and Co, London, 97 pp.

**Taxonomy**: Described as *Vespertiliochiloensis* by Waterhouse (1840: 5); currently monotypic (Novaes et al. 2018; Moratelli et al. 2019a).

**Neotype**: FMNH 24029, adult female collected by J. Vera in 1923; skull partially damaged, mandible, and skin.

**Type locality**: Cucao, Chiloé Island, Los Lagos, Chile (42°38'S, 74°06'W; sea level).

**Remarks**: The original description was based on a specimen collected in January 1836 by Lieutenant Sullivan and given to C. R. Darwin during the H. M. S. Beagle voyage. Miller and Allen (1928) were unable to locate the specimen. LaVal (1973: 43) presumed it lost and designated a neotype.

***Myotisoxyotus* (Peters, 1866)**

**Taxonomy**: Originally *Vespertiliooxyotus* Peters, 1866. We follow LaVal (1973) and Moratelli et al. (2019a) in recognizing two subspecies.

***Myotisoxyotusoxyotus* (Peters, 1866)**

Monatsberichte der Königlichen Preussische Akademie des Wissenschaften zu Berlin 1867: 16–25.

**Taxonomy**: Originally *Vespertiliooxyotus* as described by Peters (1866: 19).

**Neotype**: LACM 36878, adult female collected by D. C. Carter on July 4, 1964; skull, mandible, and skin.

**Type locality**: Gruta Rumichaca, 2 mi E La Paz, Carchi, Ecuador (00°29'N, 77°50'W; ca. 2,600 m a.s.l.).

**Remarks**: The original description was based on an adult female preserved in spirit at “Zoologischen Cabinet zu München” (Peters 1866). Presumably this “Zoologischen Cabinet” is the same as the current Zoologische Sammlung des Bayerischen Staates (= Zoologische Staatssammlung München) in Munich, Germany. LaVal (1973) noted that D. C. Carter was unable to locate the holotype there in 1966, and he pointed out that many specimens in the museum in Munich were destroyed during World War II, and must be presumed lost. Therefore, LaVal (1973: 41) designated a neotype.

***Myotisoxyotusgardneri* LaVal, 1973**

Bulletin of the Natural History Museum of Los Angeles County 15: 1–54.

**Taxonomy**: Described as *Myotisoxyotusgardneri* by LaVal (1973: 42).

**Holotype**: LSU 12924, adult male collected by A. L. Gardner on May 8, 1967; skull, mandible, baculum, and skin.

**Type locality**: Fila La Maquina, ca. 7.5 km E Canaan, San José, Costa Rica (09°27'N, 83°32'W; 2,610 m a.s.l.).

***Myotisatacamensis* (Lataste, 1892)**

Actes de la Société Scientifique du Chili 1: 70–91.

**Taxonomy**: Described as *Vespertilioatacamensis*Lataste (1892: 79); currently monotypic (Moratelli et al. 2019a).

**Neotype**: USNM 391786, adult female collected by W. Mann and S. Mann in January 1944; skull partially damaged, mandible, and skin.

**Type locality**: Near Minimini, Tarapacá, Chile (19°10'S, 69°41'W; 1,800 m a.s.l.).

**Remarks**: The original description was based on three syntypes, probably collected in February 1885 in San Pedro de Atacama, Antofagasta, Chile, and deposited at Museo Nacional de Historia Natural (Santiago, Chile), including a mounted specimen (number 277), a skull (number 1007), and a fluid preserved specimen (number 276). LaVal (1973) argued these specimens are lost or, more probable, were destroyed. Novaes et al. (2022: 3) designated a neotype.

***Myotisnesopolus* Miller, 1900**

Proceedings of the Biological Society of Washington 13: 123–127.

**Taxonomy**: Described as *Myotisnesopolus* by Miller (1900: 123). Treated as monotypic (Novaes et al. 2021a).

**Holotype**: USNM 101849, adult male collected by L. J. Guthrie on November 4, 1899; complete specimen preserved in alcohol.

**Type locality**: Near Willemstad, Curaçao, Netherlands Antilles (12°07'N, 68°52'W, ca. 35 m a.s.l.).

***Myotissimus* Thomas, 1901**

Annals and Magazine of Natural History (ser. 7) 7: 189–193.

**Taxonomy**: Described as *Myotissimus* by Thomas (1901: 541). Currently monotypic (Moratelli et al. 2011b, 2019a).

**Holotype**: BMNH 8.5.12.2, adult female collected in 1876 by W. Davis; body preserved in alcohol (skin is faded and the dorsum and venter have blocks of hair losses) with skull and mandible removed, being partially damaged.

**Type locality**: Thomas (1901) indicates Sarayacu (06°44'S, 75°06'W; Carter and Dolan 1978), Peru, as type-locality. Later, LaVal (1973) added Rio Ucayali, Loreto, Peru, 100 m a.s.l.

***Myotisdominicensis* Miller, 1902**

Proceedings of the Biological Society of Washington 15: 243–244.

**Taxonomy**: Described as *Myotisdominicensis* by Miller (1902: 243). Currently monotypic (Moratelli et al. 2019a).

**Holotype**: USNM 113564, adult male collected by H. S. Branch on July 18, 1901; body preserved in alcohol, skull and mandible removed.

**Type locality**: Island of Dominica.

***Myotisdinellii* Thomas, 1902**

Annals and Magazine of Natural History (ser. 7) 10: 493–494.

**Taxonomy**: Described as *Myotisdinellii* by Thomas (1902: 493), treated as subspecies by LaVal (1973), and as species by Miranda et al. (2013) and Moratelli et al. (2019a). Monotypic (Moratelli et al. 2019a).

**Holotype**: BMNH 0.7.9.4, adult female collected by L. Dinelli on April 7, 1899; skull severely damaged, mandible missing, and skin.

**Type locality**: Tucumán, Argentina.

***Myotiskeaysi* J.A. Allen, 1914**

Bulletin of the American Museum of Natural History 33(29): 381–389.

**Taxonomy**: Described as *Myotisruberkeaysi* by Allen (1914: 383). Monotypic (Mantilla-Meluk and Muñoz-Garay 2014; Moratelli et al. 2019a).

**Holotype**: AMNH 15814, adult male collected by H. H. Keays on December 2, 1899; skull severely damaged and skin.

**Type locality**: Inca Mines, Puno, Peru (13°30'S, 70°00'W, 1,830 m a.s.l.).

***Myotiscaucensis* J.A. Allen, 1914**

Bulletin of the American Museum of Natural History 33(29): 381–389.

**Taxonomy**: Described as *Myotiscaucensis* by Allen (1914: 386). Currently monotypic (Moratelli et al. 2013, 2019a).

**Holotype**: AMNH 32787, adult male collected by L. E. Miller on November 29, 1911; skull, mandible, and skin

**Type locality**: Rio Frío, Cauca River, Valle del Cauca, Colombia (04°09'N, 76°17'W; 1,066 m a.s.l.).

***Myotiscobanensis* Goodwin, 1955**

American Museum Novitates 1744: 1–5.

**Taxonomy**: Described as *Myotisvelifercobanensis* by Goodwin (1955: 2), but considered as a full species by de la Torre (1958) and Hall (1981). Monotypic (Moratelli et al. 2019a).

**Holotype**: AMNH 145017, adult male collected by T. Larson on June 21, 1946; skull, mandible, and skin.

**Type locality**: Cathedral at Cobán, Alta Verapaz, Guatemala (15°28'S, 90°22'W; 1,305 m a.s.l.).

***Myotisriparius* Handley, 1960**

Proceedings of the United States National Museum 112: 459–479.

**Taxonomy**: Described as *Myotissimusriparius* by Handley (1960: 466), raised to the species level by LaVal (1973). Treated as monotypic (Moratelli et al. 2019a).

**Holotype**: USNM 310255, an adult female (USNM 310255) with one embryo (7 mm crown-rump) collected by C.O. Handley and B.R. Feinstein on February 9, 1959; skull, mandible, and skin.

**Type locality**: Tacarcuna Village, Río Pucro, Darién, Panamá (07°51'N, 77°43'W, 945 m.a.s.l.).

***Myotiselegans* Hall, 1962**

University of Kansas Publications, Museum of Natural History 14(13): 161–164.

**Taxonomy**: Described as *Myotiselegans* by Hall (1962: 163). Currently monotypic (Moratelli et al. 2019a).

**Holotype**: KU 88398, adult female collected by P.L. Clifton on September 24, 1961; skull severely damaged, mandible missing, and skin.

**Type locality**: 12.5 mi N of Tihuatlán, Veracruz, Mexico (20°41'N, 97°30'W; 90 m a.s.l.).

***Myotiscarteri* LaVal, 1973**

Bulletin of the Natural History Museum of Los Angeles County 15: 1–54.

**Taxonomy**: Described as *Myotisnigricanscarteri* by LaVal (1973: 13), and elevated to the species level by Bogan (1978). Monotypic (LaVal 1973; Bogan 1978).

**Holotype**: LACM 36876, adult male collected by D. C. Carter on January 19, 1960; skull, mandible, baculum, and skin.

**Type locality**: 16 mi NE of Tamazula, Jalisco, Mexico (19°41'N, 103°14'W; 1,500 m a.s.l.).

***Myotislarensis* LaVal, 1973**

Bulletin of the Natural History Museum of Los Angeles County 15: 1–54.

**Taxonomy**: Described as full species by LaVal (1973: 44), but posteriorly treated as subspecies of *M.nesopolus* by Genoways and Williams (1979). Novaes et al. (2021a) raised *M.larensis* to species level. Monotypic (LaVal 1973; Novaes et al. 2021a).

**Holotype**: AMNH 130709, adult female collected by G. H. H. Tate on March 23, 1938; skull, mandible, and skin.

**Type locality**: Rio Tocuyo, Lara, Venezuela (10°16'N, 69°56'W; 500 m a.s.l.).

***Myotismartiniquensis* LaVal, 1973**

Bulletin of the Natural History Museum of Los Angeles County 15: 1–54.

**Taxonomy**: Described as *Myotismartiniquensis* by LaVal (1973: 35). Monotypic (Moratelli et al. 2019a).

**Holotype**: AMNH 214062, adult female collected by H. Beatty on March 15, 1967; body in alcohol, skull and mandible removed.

**Type locality**: Ca, 6 km E La Trinité, Tartane, Martinique (14°45'N, 60°54'W; ca. 65 m a.s.l.).

***Myotispilosatibialis* LaVal, 1973**

Bulletin of the Natural History Museum of Los Angeles County 15: 1–54.

**Taxonomy**: Described as *Myotiskeaysipilosatibialis* by LaVal (1973: 24), and raised to the species level by Mantilla-Meluk and Muñoz-Gray (2014). Treated as monotypic (Moratelli et al. 2019a).

**Holotype**: LACM 36879, adult male collected by R.K. LaVal and R. Valdez on July 26, 1969; skull, mandible, and skin partially damaged.

**Type locality**: 1 km W Talanga, Francisco Morazán, Honduras (14°24'N, 87°05'W; 750 m a.s.l.).

***Myotisnyctor* LaVal & Schwartz, 1974**

Caribbean Journal of Science 14: 189–192.

**Taxonomy**: Described as *Myotisnyctor* by LaVal and Schwartz (1974: 190). Currently monotypic (Moratelli et al. 2019a).

**Holotype**: KU 109473, adult male collected by D. C. Leber and A. Schwartz on February 16, 1961; skull, mandible, and skin.

**Type locality**: Cole’s Cave, Saint Thomas Parish, Barbados (13°11'N, 59°34'W; 270 m).

***Myotisfindleyi* Bogan, 1978**

Journal of Mammalogy 59(3): 519–530.

**Taxonomy**: Described as *Myotisfindleyi* by Bogan (1978: 524). Currently monotypic (Bogan 1978).

**Holotype**: USNM 512417, adult male collected by C. B. Robbins on March 14, 1976; skull, mandible, and skin.

**Type locality**: Isla Maria Magdalena, Islas Tres Marias, Nayarit, Mexico (21°27'N, 106°25'W; ca. 300 m).

***Myotisdiminutus* Moratelli & Wilson, 2011**

Mammalian Biology 76: 608–614.

**Taxonomy**: Described as *Myotisdiminutus* by Moratelli and Wilson (2011a: 609). Monotypic (Moratelli and Wilson 2011a; Moratelli et al. 2019a).

**Holotype**: USNM 58569, sub-adult male collected by D. E. Wilson on February 11, 1979; skull, mandible, and skin.

**Type locality**: Río Palenque Science Center, 47 km S (by road) from Santo Domingo, Los Rios, Ecuador (00°35'S, 79°21'W; ca. 150 m).

***Myotisizecksohni* Moratelli, Peracchi, Dias & Oliveira, 2011**

Mammalian Biology 76: 592–607.

**Taxonomy**: Described as *Myotisizecksohni* by Moratelli et al. (2011a: 597). Currently monotypic (Moratelli et al. 2011a, 2019a).

**Holotype**: ALP 6675, adult male collected by D. Dias on June 25, 2005; skull, mandible, complete post-cranial skeleton, and skin.

**Type locality**: Fazenda Maria Brandina, Tinguá Biological Reserve, Rio de Janeiro, Brazil, (22°36'S, 43°27'W; 760 m).

***Myotislavali* Moratelli, Peracchi, Dias & Oliveira, 2011**

Mammalian Biology 76: 592–607.

**Taxonomy**: Described as *Myotislavali* by Moratelli et al. (2011a: 602). Currently monotypic (Moratelli et al. 2011a, 2019a).

**Holotype**: MZUSP 18762, adult male collected by M. R. Willig on April 3, 1977; skull, mandible, and skin.

**Type locality**: 6 km S of Exu, Pernambuco State, Brazil (07°30'S, 39°43'W; 523 m).

***Myotishandleyi* Moratelli, Gardner, Oliveira & Wilson, 2013**

American Museum Novitates 3780: 1–36.

**Taxonomy**: Described as *Myotishandleyi* by Moratelli et al. (2013: 11) Currently monotypic (Moratelli et al. 2013, 2019a).

**Holotype**: USNM 370932, adult male collected by the Smithsonian Venezuela Project team on August 19, 1965; skull, mandible, and skin.

**Type locality**: Pico Ávila, 5 km northeast of Caracas, Distrito Federal, Venezuela (10°33'N, 66°52'W; 2,092 m).

***Myotismidastactus* Moratelli & Wilson, 2014**

Journal of Mammalogy 95: E17–E25.

**Taxonomy**: Described as *Myotismidastactus* by Moratelli and Wilson (2014: E19). Currently monotypic (Moratelli and Wilson 2014; Moratelli et al. 2019a).

**Holotype**: AMNH 211156, adult male collected by S. Anderson on September 9, 1965; skull, mandible, complete post-cranial skeleton, and skin.

**Type locality**: Cercado, Río Mamoré, Beni, Bolívia, ca. 23 km W of San Javier (14°34'S, 64°55'W, 160 m).

***Myotisclydejonesi* Moratelli, Wilson, Gardner, Fisher & Gutiérrez, 2016**

Special Publications, Museum of Texas Tech University 65: 49–66.

**Taxonomy**: Described as *Myotisclydejonesi* by Moratelli et al. (2016: 56). Currently monotypic (Moratelli et al. 2016, 2019a).

**Holotype**: TTU 109227, adult female collected by H. H. Genoways on January 23, 2008; skull, mandible, skin, and tissue (TK 151465).

**Type locality**: Raleigh Falls, Sipaliwini, Suriname (04°43'N, 56°12'W; 55 m).

***Myotisattenboroughi* Moratelli, Wilson, Novaes, Helgen & Gutiérrez, 2017**

Journal of Mammalogy 98: 994–1008.

**Taxonomy**: Described as *Myotisattenboroughi* by Moratelli et al. (2017: 997). Currently monotypic (Moratelli et al. 2017, 2019a).

**Holotype**: USNM 540693, adult male collected on April 4, 1981 by G. S. Morgan, L. K. Gordon and F. A. Harrington; skull, mandible, and skin.

**Type locality**: Charlottesville, 1 km N of Pirate’s Bay, Saint John Parish, Tobago Island, Republic of Trinidad and Tobago (ca. 11°19'N, 60°33'W; sea level).

***Myotisbakeri* Moratelli, Novaes, Carrión & Wilson, 2019**

Special Publications, Museum of Texas Tech University 71: 239–256.

**Taxonomy**: Described as *Myotisbakeri* by Moratelli et al. (2019b: 241). Currently monotypic (Moratelli et al. 2019b).

**Holotype**: MVZ 137909, adult male collected by M. L. Hawes on July 30, 1969; skull, mandible, and skin.

**Type locality**: 7 km SE of Chilca, Lima, Peru (12°33'S, 76°41'W; ca. 250 m).

***Myotisarmiensis* Carrión-Bonilla & Cook, 2020**

Therya 11: 508–532.

**Taxonomy**: Described as *Myotisarmiensis* by Carrión-Bonilla and Cook (2020: 515). Currently monotypic (Carrión-Bonilla and Cook 2020).

**Holotype**: MSB 262089, adult male collected by J.A. Cook and collaborators on March 20, 2012; skull, mandible, complete post-cranial skeleton, and skin.

**Type locality**: Las Nubes Ranger Station, Parque Internacional La Amistad, District of Bugaba, Province of Chiriquí, Panamá (08°53'N, 82°36'W; 2,214 m).

***Myotispampa* Novaes, Wilson & Moratelli, 2021**

Vertebrate Zoology 71: 711–722.

**Taxonomy**: Described as *Myotispampa* by Novaes et al. (2021b: 716), who considered it monotypic.

**Holotype**: AMNH 205471, adult female collected by M. D. Tuttle in January, 1963; skull, mandible, and skin.

**Type locality**: Ca. 6 km NW from Belén, Artigas, Uruguay (30°37'S, 57°50'W; 32 m elevation).

***Myotismoratellii* Novaes, Cláudio, Carrión, Abreu, Wilson, Maldonado & Weksler, 2021**

Journal of Mammalogy 103: 1–20.

**Taxonomy**: Described as *Myotismoratellii* by Novaes et al. (2021c: 10), who considered it monotypic.

**Holotype**: USNM 513482, adult male collected by A. L. Gardner on July 22, 1976; skull, mandible, and skin, all well-preserved.

**Type locality**: Vinces Canton, 3 km NE of Puerto Nuevo, Los Ríos, Ecuador (01°15'S, 78°31'W; 15 m elevation).

#### Name-bearing type specimens of species in synonymy

There are at least 29 names currently in synonymy of recognized species (Table 2). Fourteen names are junior synonyms of *M.nigricans*, three are under *M.albescens*, three under *M.chiloensis*, two under *M.ruber*, one under *M.atacamensis*, one under *M.oxyotus*, and one under *M.simus*. Below is an annotated list of these names (in chronological order), with information about the primary specimen types, their preservation, and the type localities.

**Table 2.** Names under synonymy of valid species of Neotropical *Myotis*, including information on their primary types.

Nomenclatural types	Type specimen	Synonymy	Proximal type locality
*argentatus*	Holotype KU 19228	*M.albescens*	Veracruz, Mexico
*isidori*	Holotype? MNHN 1997-1806	*M.albescens*	Corrientes, Argentina
*leucogaster*	Lectotype AMNH 385	*M.albescens*	Bahia, Brazil
*punensis*	Holotype AMNH 36263	*M.albescens*	Guayas, Ecuador
*nicholsoni*	Holotype FMNH 50783	*M.atacamensis*	Arequipa, Peru
*aelleni*	Holotype MHNG 1486.76	*M.chiloensis*	Chubut, Argentina
*arescens*	Holotype FMNH 24396	*M.chiloensis*	Valparaiso, Chile
*gayi*	Not located	*M.chiloensis*	Los Lagos, Chile
*alter*	Holotype BMNH 0.6.29.23	*M.levis*	Paraná, Brazil
*nubilus*	Holotype? ZSM 121	*M.levis*	Southern Brazil
*polythrix*	Syntypes MNHN 842, 843	*M.levis*	Rio Grande do Sul, Brazil
*arsinoe*	Holotype RNH 17635	*M.nigricans*	Suriname
*bondae*	Holotype AMNH 14587	*M.nigricans*	Santa Marta, Colombia
*brasiliensis*	Not located	*M.nigricans*	Brazil
*chiriquensis*	Holotype AMNH 18736	*M.nigricans*	Chiriquí, Panama
*concinnus*	Syntypes ANSP 1114, 1115	*M.nigricans*	San Salvador, El Salvador
*dalquesti*	Holotype KU 23839	*M.nigricans*	Veracruz, Mexico
*esmeraldae*	Holotype AMNH 33239	*M.nigricans*	Esmeraldas, Ecuador
*exiguus*	Holotype ANSP 5626	*M.nigricans*	Panamá, Panama
*hypothrix*	Holotype? MNHN 1903-41	*M.nigricans*	Beni, Bolivia
*maripensis*	Holotype AMNH 17069	*M.nigricans*	Bolívar, Venezuela
*mundus*	Holotype ANSP 1829	*M.nigricans*	Zulia, Venezuela
*parvulus*	Lectotype RNH 17621	*M.nigricans*	Brazil
*spixi*	Not located	*M.nigricans*	Brazil
*splendidus*	Holotype? ZSM 142	*M.nigricans*	US Virgin Islands
*thomasi*	Not located	*M.oxyotus*	Napo, Ecuador
*cinnamomeus*	Not located	*M.ruber*	Paraguay
*kinnamon*	Holotype? MNHN 1997-2056	*M.ruber*	Minas Gerais, Brazil
*guaycuru*	Holotype ALP 9277	*M.simus*	Mato Grosso do Sul, Brazil

***Vespertilioleucogaster* Schinz, 1821**

In “Das tierreich eingetheilt nach dem Bau der thiere als Grundlage ihrer Naturgeschichte und der vergleichenden Anatomie von dem Herrn Ritter von Cuvier, volume 1”. Saugethiere und Vögel, Stuttgart and Tübingen, 894 pp.

**Taxonomy**: Described as *Vespertilioleucogaster* (currently allocated to *Myotis*) by Schinz (1821: 180). Currently a junior synonym of *Myotisalbescens* (Miller and Allen 1928; LaVal 1973; Wilson 2008).

**Lectotype**: AMNH 385, adult (undetermined sex) collected by Maximilian, Prinz zu Wied-Neuwied (date not specified); taxidermized skin and skull not removed (see Avila-Pires 1965).

**Type locality**: Mucurí, Bahia, Brazil.

***Vespertiliobrasiliensis* Spix, 1823**

In “Simiarum et Vespertilionum brasiliensium species novae (…)”. Typis Francisci Serephici Hübschmanni, Monaco, xvi + 72 pp.

**Taxonomy**: Described as *Vespertiliobrasiliensis* (currently allocated to *Myotis*) by Spix (1823: 63). Currently a junior synonym of *Myotisnigricans* (Miller and Allen 1928; Wilson 2008).

**Type specimen**: No specimen was designated by the author. Just like Carter and Dolan (1978), we have not found any reference specimens deposited in European collections.

**Type locality**: Brazil.

**Remarks**: The original name combination is preoccupied by *Vespertiliobrasiliensis* Desmarest, 1822 (currently *Eptesicusbrasiliensis*), hence, Fischer (1829) replaced it by *Vespertiliospixii*.

***Vespertiliopolythrix* I. Geoffroy, 1824**

Annales de Sciences Naturelles de Paris 3: 440–447.

**Taxonomy**: Described as *Vespertiliopolythrix* (currently allocated to *Myotis*) by Geoffroy Saint-Hilaire (1824: 443). Currently a junior synonym of *Myotislevis* (LaVal 1973; Wilson 2008).

**Syntypes**: MNHN 842 (adult, undetermined sex), MNHN 843 (adult male), ZMB 3911 (adult, undetermined sex) collected by A. Geoffroy Saint-Hilaire, date not specified. All specimens are skins taxidermized (faded) with skull not removed.

**Type locality**: Rio Grande do Sul or Minas Gerais, Brazil.

**Remarks**: According to Turni and Kock (2008), the name *polythrix* is a nomen oblitum, due to page priority. This is the first available name (p. 443), whereas *levis* (nomen protectum) is on page 444 in Geoffroy’s publication (1824).

***Vespertiliospixii* Fischer, 1829**

In “Synopsis mammalium”. Stuttgardtiae: J. G. Cottae, xlii + 752 pp.

**Taxonomy**: This name was proposed in replacement for *Vespertiliobrasiliensis* Spix, 1823, considering that this name was preoccupied by *Vespertiliobrasiliensis* Desmarest, 1822 (= *Eptesicusbrasiliensis*). Currently a junior synonym of *Myotisnigricans* (Miller and Allen 1928; Cabrera 1958; Wilson 2008).

***Vespertilioparvulus* Temminck, 1840**

In “Monographies de mammalogie ou description de quelques genres de mammifères dont les espèces ont été observées dans les différens musées de l’Europe”. E. d’Ocagne et A. Bertrand, Paris, 141–272.

**Taxonomy**: Described as *Vespertilioparvulus* (currently allocated to *Myotis*) by Temminck (1840: 246). Currently a junior synonym of *Myotisnigricans* (Miller and Allen 1928; LaVal 1973; Wilson 2008).

**Lectotype**: RNH 17621, adult, sex undetermined, collected by J. Natterer (date not specified); skull severely damaged and skin faded.

**Type locality**: Brazil.

***Vespertilioarsinoe* Temminck, 1840**

In “Monographies de mammalogie ou description de quelques genres de mammifères dont les espèces ont été observées dans les différens musées de l’Europe”. E. d’Ocagne et A. Bertrand, Paris, 141–272.

**Taxonomy**: Described as *Vespertilioarsinoe* (currently allocated to *Myotis*) by Temminck (1840: 247). Currently a junior synonym of *Myotisnigricans* (LaVal 1973; Wilson 2008).

**Holotype**: RNH 17635, adult female (collector and date of capture are unknown); skull partially damaged and skin faded.

**Type locality**: Surinam.

***Vespertiliohypothrix* d’Orbigny & Gervais, 1847**

In “Voyage dans l’Amérique méridionale (…). P. Bertrand/Strasbourg: V. Levrault, Paris 4: 1–32.

**Taxonomy**: Described as *Vespertiliohypothrix* (currently allocated to *Myotis*) by d’Orbigny and Gervais (1847: 14). Currently a junior synonym of *Myotisnigricans* (Miller and Allen 1928; Cabrera 1958; Wilson 2008).

**Holotype**: MNHN AC 1903-41, sex, age, collector, and date undetermined; stretched skin only.

**Type locality**: Moxos [Beni], Bolivia.

***Vespertilioisidori* d’Orbigny & Gervais, 1847**

In “Voyage dans l’Amérique méridionale (…)”. P. Bertrand/Strasbourg: V. Levrault, Paris 4: 1–32.

**Taxonomy**: Described as *Vespertilioisidori* (currently allocated to *Myotis*) by d’Orbigny and Gervais (1847: 16). Currently a junior synonym of *Myotisalbescens* (Miller and Allen 1928). However, based on observations made by Carter and Dolan (1978), Wilson (2008) did not include this name in the synonym list for *M.albescens* (see discussion in Remarks section)

**Holotype**: Probably MNHN 1997-1806, adult, sex undetermined; skull (damaged), mandible, and skin.

**Type locality**: Corrientes, Argentina.

**Remarks**: Rode (1941) indicated the specimen MNHN 865 as the holotype. However, Carter and Dolan (1978) show that there was confusion when interpreting a Cadre number, used to guide visitors about a specimen on display in the museum, with the catalog number. Thus, Carter and Dolan indicate that this is not the type specimen of this name and have not found any other specimen in collections in Europe that could be. One of us (RM) found the supposed specimen used for the description by d’Orbigny and Gervais deposited in the mammal collection of the Muséum National D’Histoire Naturelle, Paris, France. The presumable holotype (MNHN 1997-1806) is an adult (sex undetermined). The skull reassembles *M.albescens*, but the color pattern of the skin is not a Neotropical *Myotis*.

***Vespertiliosplendidus* Wagner, 1855**

In “Die säugthiere in abbildungen nach der natur mit beschreibungen von Dr. Johann Christian Daniel von Schreber (…)”. T.O. Weigel, Leipzig, xxvi + 810 pp.

**Taxonomy**: Described as *Vespertiliosplendidus* (currently allocated to *Myotis*) by Wagner (1855: 148). Currently a junior synonym of *Myotisnigricans* (see Carter and Dolan 1978; Wilson 2008).

**Holotype**: ZSM 142, adult of undetermined sex, probably collected by A. F. W. Schimper (date not specified); skin only, slightly faded.

**Type locality**: St. Thomas [American Virgin Islands (Carter and Dolan 1978)].

***Vespertilionubilus* Wagner, 1855**

In “Die säugthiere in abbildungen nach der natur mit beschreibungen von Dr. Johann Christian Daniel von Schreber (…)”. T. O. Weigel, Leipzig, xxvi + 810 pp.

**Taxonomy**: Described as *Vespertilionubilus* (currently allocated to *Myotis*) by Wagner (1855: 752). Currently a junior synonym of *Myotislevis* (see LaVal 1973; Wilson 2008).

**Holotype**: ZSM 121, subadult, sex undetermined; collector and date of capture are unknown; skin taxidermized with skull not removed.

**Type locality**: Brazil.

***Vespertiliocinnamomeus* Wagner, 1855**

In “Die säugthiere in abbildungen nach der natur mit beschreibungen von Dr. Johann Christian Daniel von Schreber (…)”. T. O. Weigel, Leipzig, xxvi + 810 pp.

**Taxonomy**: Wagner (1855: 755) proposed the name *Vespertiliocinnamomeus* as a substitute for *Vespertilioruber* É. Geoffroy, 1806 believing that “chauve-sourris cannelle” from Azara (1801) was a *Noctilio* Linnaeus, 1766. However, Miller and Allen (1928) resolved Wagner’s misunderstanding, indicating that both the name *ruber* and *cinnamomeus* were based on the same specimen. Currently a junior synonym of *Myotisruber* (see Miller and Allen 1928; LaVal 1973; Wilson 2008).

***Vespertiliokinnamon* Gervais, 1856**

In “Animaux nouveaux ou rares recueillis pendant l’expédition dans les parties centrales de l’Amérique du Sud (…)”. P. Bertrand, Paris, 25–88.

**Taxonomy**: Described as *Vespertiliokinnamon* (currently allocated to *Myotis*) by Gervais (1856: 84). Currently a junior synonym of *Myotisruber* (Miller and Allen 1928; Cabrera 1958).

**Holotype**: MNHN 1997-2056, adult male collected on 1844 (collector not specified); skin only.

**Type locality**: Capela Nova, Minas Gerais, Brazil.

***Vespertiliomundus* H. Allen, 1866**

Proceedings of the Academy of Natural Sciences of Philadelphia 18: 279–288.

**Taxonomy**: Described as *Vespertiliomundus* (currently allocated to *Myotis*) by Allen (1866: 280). Currently a junior synonym of *Myotisnigricans* (Miller and Allen 1928; LaVal 1973; Wilson 2008).

**Holotype**: ANSP 1829 (=USNM 5547), subadult female collected by S. Hayes (date not specified), currently deposited in the mammal collection of the Academy of Natural Sciences of Drexel University (Philadelphia, USA); complete specimen preserved in alcohol with skin faded.

**Type locality**: Maracaibo, Venezuela.

***Vespertilioconcinnus* H. Allen, 1866**

Proceedings of the Academy of Natural Sciences of Philadelphia 18: 279–288.

**Taxonomy**: Described as *Vespertilioconcinnus* (currently allocated to *Myotis*) by Allen (1866: 281). Currently a junior synonym of *Myotisnigricans* (Miller and Allen 1928; Cabrera 1958; LaVal 1973; Wilson 2008).

**Syntypes**: ANSP 1114 and ANSP 1115, are adult females, collected by J. Leidy (date not specified); Body preserved in alcohol with skin faded, skull and mandible removed.

**Type locality**: San Salvador, El Salvador.

***Vespertilioexiguus* H. Allen, 1866**

Proceedings of the Academy of Natural Sciences of Philadelphia 18: 279–288.

**Taxonomy**: Described as *Vespertilioexiguus* (currently allocated to *Myotis*) by Allen (1866: 281). Currently a junior synonym of *Myotisnigricans* (Miller and Allen 1928; Cabrera 1958).

**Holotype**: ANSP 5626 (= USNM 5373), adult female collected by S. Hayes (date not specified) is currently deposited in the mammal collection of the Academy of Natural Sciences of Drexel University (Philadelphia, USA); complete specimen preserved in alcohol with skin faded.

**Type locality**: Aspinwall, NG. (= Colón, Panama).

***Vespertiliogayi* Lataste, 1892**

Actes de la Société Scientifique du Chili 1: 70–91.

**Taxonomy**: Described as *Vespertiliogayi* (currently allocated to *Myotis*) by Lataste (1892: 79), currently considered a junior synonym of *Myotischiloensis* (Miller and Allen 1928; Cabrera 1958; Wilson 2008).

**Type specimen**: None. We did not access the original publication describing the species. However, no specimens from Lataste that could match the description of *M.gayi* are available in collections in Europe or South America. Probably, these specimens are lost.

**Type locality**: Valdivia, Chile.

***Myotisthomasi* Cabrera, 1901**

Boletín de la Sociedad Española de Historia Natural 1: 367–373.

**Taxonomy**: Described as *Myotisthomasi* by Cabrera (1901: 370). Currently a junior synonym of *Myotisoxyotus* (Miller and Allen 1928; LaVal 1973; Wilson 2008).

**Type specimen**: None. The original description was based on an adult female preserved in alcohol that, according to the author, was deposited in the mammal collection of the Museo Nacional de Ciencias Naturales (Madrid, Spain). However, a voucher number for the specimen was not listed by Cabrera (1901). Nevertheless, Carter and Dolan (1978) did not find the representative specimen in the MNCN collection and suspect that when moving to Argentina, A. Cabrera would have taken the type specimens. There are no specimens of *Myotis* that can represent the type of *M.thomasi* in the Museo de La Plata (Itatí Olivares, pers. comm.). We presume that probably this type specimen is lost.

**Type locality**: In the original description, Cabrera (1901) argued that he did not have reliable data on the geographical origin of the specimen, but that it was probably from southern Brazil. Later, Cabrera (1902) corrected this to “Archidona [sobre el citado río], Napo, Ecuador”.

***Myotischiriquensis* J.A. Allen, 1904**

Bulletin of the American Museum of Natural History 20: 29–80.

**Taxonomy**: Described as *Myotischiriquensis* by Allen (1904: 77). Currently a junior synonym of *Myotisnigricans* (Miller and Allen 1928; Cabrera 1958; LaVal 1973; Wilson 2008).

**Holotype**: AMNH 18736, adult female, collected by J. H. Batty on October 16, 1901; skull, mandible, and skin.

**Type locality**: Boquerón, Chiriquí, Panama.

***Myotispunensis* J.A. Allen, 1914**

Bulletin of the American Museum of Natural History 33(29): 381–389.

**Taxonomy**: Described as *Myotispunensis* by Allen (1914: 383). Currently a junior synonym of *Myotisalbescens* (see Moratelli and Wilson 2011b).

**Holotype**: AMNH 36263, sub-adult male collected by W.B. Richardson on May 8, 1913; skull (partially damaged), mandible, and skin.

**Type locality**: Isla Puna, Guayaquil, Guayas, Ecuador.

***Myotisbondae* J.A. Allen, 1914**

Bulletin of the American Museum of Natural History 33(29): 381–389.

**Taxonomy**: Described as *Myotisbondae* by Allen (1914: 384). Currently a junior synonym of *Myotisnigricans* (Miller and Allen 1928; Cabrera 1958; LaVal 1973; Wilson 2008).

**Holotype**: AMNH 14587, adult of undetermined sex, collected by H. H. Smith in June 1898; skull, mandible, and skin.

**Type locality**: Bonda, Santa Marta, Colombia.

***Myotismaripensis* J.A. Allen, 1914**

Bulletin of the American Museum of Natural History 33(29): 381–389.

**Taxonomy**: Described as *Myotismaripensis* by Allen (1914: 385). Currently a junior synonym of *Myotisnigricans* (Miller and Allen 1928; Cabrera 1958; LaVal 1973; Wilson 2008).

**Holotype**: AMNH 17069, adult female collected by S. M. Klages on December 13, 1909; skull, mandible, and skin.

**Type locality**: Maripa, Venezuela.

***Myotisesmeraldae* J.A. Allen, 1914**

Bulletin of the American Museum of Natural History 33(29): 381–389.

**Taxonomy**: Described as *Myotisesmeraldae* by Allen (1914: 385). Currently a junior synonym of *Myotisnigricans* (Miller and Allen 1928; Cabrera 1958; Wilson 2008).

**Holotype**: AMNH 33239, adult male, collected by W. B. Richardson on November 5, 1912; skull, mandible, and skin.

**Type locality**: Esmeraldas, Ecuador.

***Myotischiloensisalter* Miller & Allen, 1928**

Bulletin of the United States National Museum 144: 1–218.

**Taxonomy**: Described as a subspecies of *Myotischiloensis* by Miller and Allen (1928). Currently a junior synonym of *Myotislevis* (LaVal 1973; Wilson 2008).

**Holotype**: BMNH 0.6.29.23, adult female collected by G. Grillo (date not specified); body in alcohol, skull and mandible removed.

**Type locality**: Palmeira, Paraná, Brazil.

***Myotisnigricansnicholsoni* Sanborn, 1941**

Field Museum of Natural History, Zoological Series 27: 371–387.

**Taxonomy**: Described as a subspecies of Myotisnigricans by Sanborn (1941: 382). Currently a junior synonym of *Myotisatacamensis* (LaVal 1973; Wilson 2008).

**Holotype**: FMNH 50783, adult male collected by C. C. Sanborn on October 17, 1939; skull, mandible, and skin, all well-preserved.

**Type locality**: Hacienda Chucarapi, Tambo Valley, Arequipa, Peru.

***Myotischiloensisarescens* Osgood, 1943**

Field Museum of Natural History, Zoological Series 30: 1–268.

**Taxonomy**: Described as a subspecies (Osgood 1943: 55), but currently considered a junior synonym of *Myotischiloensis* (LaVal 1973; Wilson 2008).

**Holotype**: FMNH 24396, adult male collected by J. A. Wolffsohn on January 1, 1925; skin only.

**Type locality**: Hacienda Limache, Valparaíso, Chile.

***Myotisguaycuru* Proença, 1943**

Revista Brasileira de Biologia 3: 313–315.

**Taxonomy**: Described as *Myotisguaycuru* by Proença (1943: 314), but currently considered a junior synonym of *Myotissimus* (Wilson 2008; Moratelli et al. 2011b).

**Holotype**: ALP 9277, an adult female collected in 1940 by Scientific Committee of the Oswaldo Cruz Institute, headed by L. Travassos; body preserved in fluid (severely damaged), with the skull (including mandible) removed and complete.

**Type locality**: Rio Miranda, Salobra, Mato Grosso do Sul, Brazil.

***Myotisargentatus* Dalquest & Hall, 1947**

University of Kansas Publications, Museum of Natural History 1(12): 237–244.

**Taxonomy**: Described as a full species by Dalquest and Hall (1947: 239). Currently a junior synonym of *Myotisalbescens* (see LaVal 1973).

**Holotype**: KU 19228, adult male collected by W. W. Dalquest on February 2, 1947; skull, mandible, and skin.

**Type locality**: 14 km SW of Coatzocoalcos, Veracruz, Mexico (30 m elevation).

***Myotisnigricansdalquesti* Hall & Alvarez, 1961**

University of Kansas Publications, Museum of Natural History 14(4): 69–72.

**Taxonomy**: Described as a subspecies of *Myotisnigricans* by Hall and Alvarez (1961: 71), but currently considered a junior synonym of *Myotisnigricans* (LaVal 1973).

**Holotype**: KU 23839, adult male collected by W. W. Dalquest on January 5, 1948; skull, mandible, and skin.

**Type locality**: 3 km E of San Andrés Tuxtla, Veracruz, Mexico (304 m elevation).

***Myotisaelleni* Baud, 1979**

Revue Suisse de Zoologie 86(1): 267–278.

**Taxonomy**: Described as a full species by Baud (1979: 268), but currently considered a junior synonym of *Myotischiloensis* (Novaes et al. 2018).

**Holotype**: MHNG 1486.76, adult male collected by A. Kovacs on December 19, 1975; body preserved in alcohol, skull and mandible removed.

**Type locality**: El Hoyo de Epuyen, 42°10'S, 71°21'W (230 m elevation), Provincia de Chubut, Argentina.

## ﻿Discussion

*Myotis* is the most speciose bat genus in the Neotropics, with 33 species recognized currently (Bogan 1978; Moratelli et al. 2019a, b; Novaes et al. 2021a, b, c). Several species’ descriptions and revalidations have been recently proposed (e.g., LaVal 1973; Moratelli et al. 2011a, 2013, 2016, 2017, 2019b; Novaes et al. 2018, 2021a, b, c), and the evidence available points in the direction of hidden diversity (Clare et al. 2007; Larsen et al. 2012; Novaes et al. 2018; Carrión-Bonilla and Cook 2020). In this scenario of intense taxonomic change, a careful assessment of all name-bearing types is essential to the correct application of names to newly identified lineages and other nomenclatural acts. Below, we point out some nomenclatural issues still associated with name-bearing types of Neotropical *Myotis*.

Since its description, *Myotisnigricans* has been treated as a widely distributed species, and several subspecies have been recognized by different authors. However, recent studies have merged evidence indicating that *M.nigricans* is composite, as currently recognized, representing a complex of allopatric species (Moratelli and Wilson 2011a; Moratelli et al. 2011a, 2016, 2017, 2019b; Novaes et al. 2021b). The name *nigricans* seems to apply to Atlantic Forest populations from southeastern Brazil and southern South America, considering the type locality (Moratelli and Wilson 2011a; Moratelli et al. 2013, 2017). Therefore, it is necessary to reassess the taxonomic status of populations from tropical Mexico, Central America, and northern South America currently recognized as *M.nigricans* (and its subspecies). In this case, names currently treated under synonymy might apply to these potential new taxa.

Currently, 14 names are under synonymy of *M.nigricans* and can be available to use after a careful taxonomic review that considers the examination of type specimens. An example is the name *Vespertiliosplendidus* Wagner, 1855 (= *Myotissplendidus*), described based on a specimen from “St. Thomas” (Wagner 1855). Carter and Dolan (1978) indicated the type locality as “St. Thomas [American Virgin Islands]”, which was followed by subsequent authors (e.g., Wilson 2008). However, *Myotis* apparently does not occur either on the US Virgin Islands (Bacle et al. 2008) or on the nearest Caribbean islands (Puerto Rico, British Virgin Islands, Anguilla; Timm and Genoways 2003; Genoways et al. 2007). On the other hand, “St. Thomas” is a locality on the Caribbean Island of Barbados, where *M.nyctor* is the only species known to occur (Novaes et al. 2021a). Based on this scenario, *Myotissplendidus* is a very rare (or extinct) species (and unique representative of the genus) on the US Virgin Islands; or the geographical origin of the holotype of *Myotissplendidus* is Barbados, not US Virgin Islands, and the name is the senior synonym of *Myotisnyctor*. In any case, *Myotissplendidus* is unlikely to be a synonym for *Myotisnigricans*, considering the biogeographical history of colonization of the Caribbean, where each island has its own unique species of *Myotis*, and there is no evidence of the occurrence of *M.nigricans* as recognized by Moratelli et al. (2017) and Novaes et al. (2021a).

Another important issue is the validity of some names occasionally found in the literature on *Myotis* taxonomy. In their catalogue of type specimens of neotropical bats deposited in selected European museums, Carter and Dolan (1978) listed “*Vespertiliocarbonarius* Wagner” based on a specimen (ZSM 124) from Brazil obtained by J.F. Brandt, whose label reads “*Vespertiliocarbonarius* Wagn. / 1843 / Brandt / Brasil”. This specimen (taxidermized skin with skull not removed) was examined by us and it resembles *M.riparius* in size and the fur texture, length, and coloration. However, as with Carter and Dolan (op cit.), we were also unable to locate the publication with the species description. It is not impossible that this name was formally published (considering the vast, and sometimes rare, production of Wagner). However, if a publication containing the species description is found, we suggest that *Vespertiliocarbonarius* should be treated as a *nomen oblitum*, following article 23.9.1 from ICZN (1999).

For another example, Cornalia (1849) assigned the name “*Vespertilioquixensis* Osculati” to the synonymy of *Vespertilioosculati* (= *Myotisnigricansosculati*). In an introduction to the facsimile reprint of Cornalia’s (1849) publication, Cagnolaro and Violani (1988) recommended treating *V.quixensis* as a *nomen nudum*, but the name became available in the combination *Phyllostomusquixensis* Osculati, 1854: 53. It is possible that the description of *quixensis* appeared in the first edition of the Osculati’s publication; however, we have not been able to examine that publication due to its rarity.

*Myotis* comprises a diverse group in number of species compared to other neotropical bat genera. However, its species richness does not reflect its phenotypic diversity, characterized by a low morphological differentiation (Ghazali et al. 2017; Moratelli et al. 2019a). Due to the large number of species names proposed, this catalogue puts together information on name-bearing types of species treated as valid or under synonymy as an aid for future taxonomic works.
